# Fulminant amebic colitis complicated by appendiceal perforation and massive abdominal hemorrhage: a case report and literature review

**DOI:** 10.3389/fmed.2026.1760895

**Published:** 2026-02-19

**Authors:** Xin Zhang, Wenxiong Li, Song Zhao, Yue Han, Wenliang Ma, Zhaoyang Jiang, Youwei Ma, Guichen Zhang, Jingyi Wang, Huimiao Jia, Shuyan Guo, Na Cui

**Affiliations:** 1 Department of Critical Care Medicine, Beijing Tuberculosis and Thoracic Tumor Research Institute, Beijing Chest Hospital, Capital Medical University, Beijing, China; 2 Department of Critical Care Medicine, Beijing Chao-Yang Hospital, Capital Medical University, Beijing, China

**Keywords:** bowel perforation, *Entamoeba histolytica*, fulminant amebic colitis, intra-abdominal hemorrhage, metagenomic next-generation sequencing

## Abstract

**Introduction:**

Fulminant amebic colitis complicated by intestinal perforation or massive intra-abdominal hemorrhage is uncommon but associated with extremely high mortality. In non-endemic regions, diagnosis is frequently delayed because early manifestations resemble bacterial appendicitis or perforated peritonitis.

**Case presentation:**

We report a fatal case of *Entamoeba histolytica* infection presenting with appendiceal perforation, septic shock, and recurrent intra-abdominal bleeding. Surgery revealed extensive transmural necrosis, deep ulcers, and exposure of submucosal vessels. Metagenomic next-generation sequencing (mNGS) of blood and peritoneal drainage fluid was performed, followed by histopathological confirmation. Despite emergent appendectomy, bowel resection, and prompt initiation of anti-amebic therapy, the patient developed refractory septic shock and recurrent intra-abdominal hemorrhage, resulting in death.

**Conclusion:**

mNGS can facilitate early recognition of severe amebiasis when conventional diagnostic modalities are uncertain, particularly in non-endemic settings. Fulminant amebic colitis complicated by perforation or hemorrhage carries a poor prognosis. Timely clinical suspicion and early anti-amebic therapy are essential to improve outcomes.

## Introduction

Amebiasis occurs worldwide, with the highest burden occurring in the low-income regions of tropical and subtropical countries. Intestinal amebiasis, caused by the protozoan *Entamoeba histolytica*, has a broad clinical spectrum ranging from asymptomatic colonization to fulminant necrotizing colitis. Appendicitis is an uncommon manifestation, and clinicians in non-endemic areas often maintain a low index of suspicion for amebic infections. Conventional diagnostic methods for amebiasis include stool microscopy and antigen detection, polymerase chain reaction (PCR), serological testing, and histopathological examination. These targeted tests remain the primary means of diagnosing typical intestinal disease. However, in non-endemic areas and in patients presenting with early, atypical, or extraintestinal manifestations, these tests may not be readily available or may yield false-negative results. In such cases, metagenomic next-generation sequencing (mNGS) can complement traditional diagnostic methods by providing unbiased multi-pathogen detection and etiological clues from the sample.

Herein, we report a rare case of histopathologically confirmed intestinal amebiasis complicated by appendiceal perforation and sepsis. This case highlights the diagnostic value of mNGS in nonendemic settings and provides a concise analysis of the clinical features associated with this severe presentation.

## Case presentation

**Figure 1:**
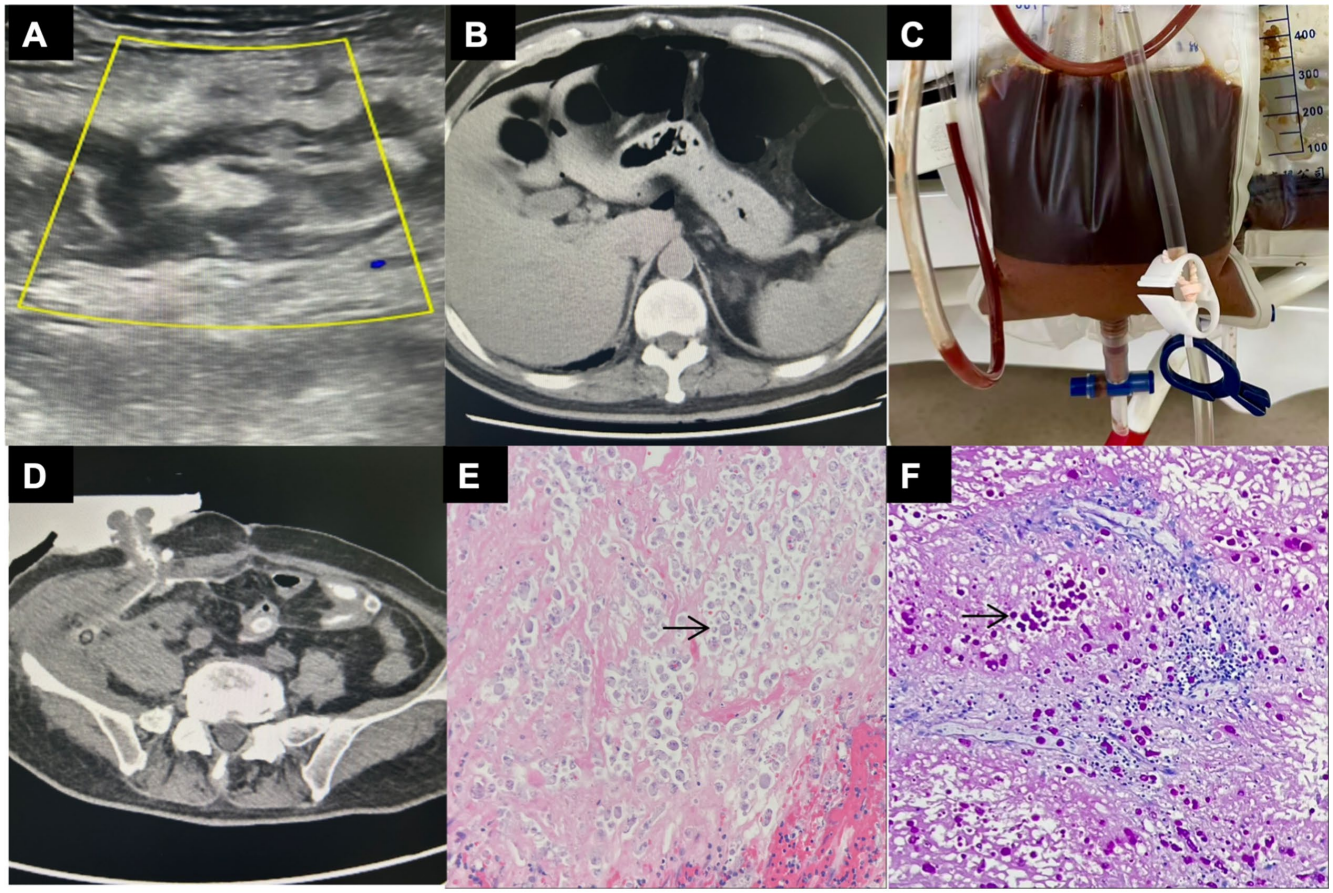
Imaging findings, drainage fluid characteristics, and pathological features. **(A)** Abdominal ultrasonography showing a heterogeneous hypoechoic lesion in the right lower abdomen measuring 9.8 × 3.8 cm with poorly defined margins. **(B)** Emergency abdominal CT demonstrating pneumoperitoneum and colonic dilation with intraluminal gas, consistent with intestinal perforation. **(C)** Turbid, dark reddish peritoneal drainage fluid with obvious layering. **(D)** CT scan shows a high-density shadow in the abdominal drainage tube, suggesting contrast agent extravasation and possible intestinal fistula. **(E)** H&E staining shows trophozoites of *E. histolytica* (arrow) with ingested erythrocytes. **(F)** PAS staining further accentuates the trophozoites. The arrow in the figure highlights an *Entamoeba histolytica* trophozoite, characterized by its round morphology and histiocyte-like appearance. Ingested erythrocytes are visible within the cytoplasm, and the organism exhibits a small, round nucleus with an eccentric position.

A 59-year-old man with a history of type 2 diabetes mellitus who was treated with liraglutide presented to the gastroenterology clinic in early September with abdominal pain in the right lower quadrant. Initial laboratory tests showed leukocytosis (11.28 × 10^9^/L) with neutrophilia (8.25 × 10^9^/L) and an elevated C-reactive protein level of 1.44 mg/dL. Abdominal computed tomography (CT) revealed an indistinct appendix with surrounding fat stranding and small mesenteric lymph nodes. Abdominal ultrasonography revealed increased echogenicity in the adjacent omentum and pericecal fluid. Acute appendicitis was suspected, and cefotiam (2 g twice daily) was initiated; however, the patient experienced no symptom relief. On September 20, a repeat CT revealed acute appendicitis with a pericecal abscess. Ultrasound showed a heterogeneous lesion in the right lower abdomen measuring 9.8 × 3.8 cm with poorly defined margins (Figure 1A). The patient was then admitted to the surgical department.

On admission, his vital signs were stable, and abdominal examination revealed focal tenderness at the McBurney’s point. Laboratory tests showed worsening leukocytosis (15.52 × 10^9^/L) and neutrophilia (12.77 × 10^9^/L). On September 25, ultrasonography-guided percutaneous drainage yielded a small amount of reddish-brown purulent fluid, which was not subjected to microbiological testing. The antimicrobial therapy was escalated to cefoperazone–sulbactam (3 g every 12 h). Despite therapy, his abdominal pain persisted, and he developed intermittent fever up to 38.5 °C.

On October 1, he experienced sudden diffuse abdominal pain, high fever (38.7 °C), tachycardia, and hypotension (nadir 88/56 mmHg). Emergency CT revealed a pneumoperitoneum and colonic dilation with intraluminal gas (Figure 1B), consistent with gastrointestinal perforation and septic peritonitis. The patient underwent emergency debridement of the periappendiceal abscess. Intraoperatively, large amounts of purulent material were noted and a 1 cm perforation was identified on the cecal wall. He was admitted to the intensive care unit (ICU) postoperatively and treated with piperacillin–tazobactam. A culture of the peritoneal fluid showed *Enterococcus faecium*. mNGS detected *E. faecium* (27,018 reads, 77.74%) and *Entamoeba histolytica* (19 reads, 80.12%) in the peritoneal fluid and *E. faecium* (eight reads, 14.82%) in the blood. The following day, the patient was extubated and transferred to the general ward, where he continued to have an intermittent fever.

On October 7, the patient developed recurrent abdominal pain with tachypnea and shock (heart rate, 120 beats/min; blood pressure, 95/65 mmHg). The abdominal drain produced large volumes of turbid, dark reddish fluid with obvious layering (Figure 1C), and amylase levels reached 11,353 U/L. CT demonstrated contrast agent extravasation, suggesting an intestinal fistula (Figure 1D). Given the possibility of gastrointestinal perforation, emergency exploratory laparotomy was performed. Intraoperatively, approximately 400 mL of purulent fluid was found, with marked dilatation of the small intestine and a thick layer of fibrinous exudate coating the surface. Approximately 40 cm proximal to the ileocecal region, the ileum showed gangrenous changes, with extremely fragile bowel walls that ruptured upon gentle palpation. The ileocecal area appeared anatomically disorganized, and the cecum extending to approximately 10 cm of the ascending colon was involved in severe infection, appearing friable and densely adherent to the omentum. A ruptured abscess cavity was observed lateral to the appendiceal base, extending into the retroperitoneum and psoas muscle. Ileocecal resection and right hemicolectomy with end ileostomy were performed, followed by copious irrigation with normal saline solution. Postoperatively, the patient was admitted to the ICU and was treated with linezolid and meropenem. Postoperative cultures of peritoneal drainage fluid grew *Escherichia faecium*, *Escherichia coli*, and *Candida glabrata*. Blood cultures remained negative. His procalcitonin level decreased from 2.48 ng/mL to 0.46 ng/mL. He was extubated on postoperative day 5 but continued to experience intermittent fever, and caspofungin was added.

On October 24, the patient developed septic shock and respiratory failure (HR, 120 beats/min; BP, 86/48 mmHg; RR, 30 breaths/min) requiring intubation and ICU readmission. Blood mNGS identified *Klebsiella pneumoniae* (5,808 reads), *Pseudomonas aeruginosa* (121 reads), *Candida albicans* (9 reads), *C. glabrata* (2 reads), and *E. histolytica* (12 reads, 73.71%). Anti-amebic therapy with intravenous metronidazole (0.5 g) was initiated every 8 h, along with ceftazidime–avibactam (2.5 g every 8 h). Histopathological examination of the resected ileocecal specimens revealed extensive mucosal ulceration and necrosis with abscess formation, cecal perforation, transmural acute and chronic inflammation, vascular congestion, and a subserosal abscess. Histopathological examination of the resected ileocecal specimens with hematoxylin–eosin (H&E) staining revealed extensive inflammation and necrosis (Figure 1E). Additional periodic acid–Schiff (PAS) staining highlighted trophozoites with ingested erythrocytes (Figure 1F). Upon further questioning, the patient reported recent residence in Africa, the Middle East, and Southeast Asia with preceding diarrhea, consistent with amebiasis.

On November 4, the patient developed active abdominal wall bleeding with hemorrhagic shock (HR, 130 beats/min; blood pressure, 75/40 mmHg; and hemoglobin, 24 g/L). Emergency laparotomy revealed massive intraabdominal bleeding and chronic oozing at the stomatal site. Hemostasis was achieved using debridement and packing, followed by ICU care. Although he stabilized temporarily, recurrent bleeding occurred on November 13, with hemoglobin level decreasing to 50 g/L. The family declined further surgical intervention, and the patient died on November 14.

**Figure 2:**
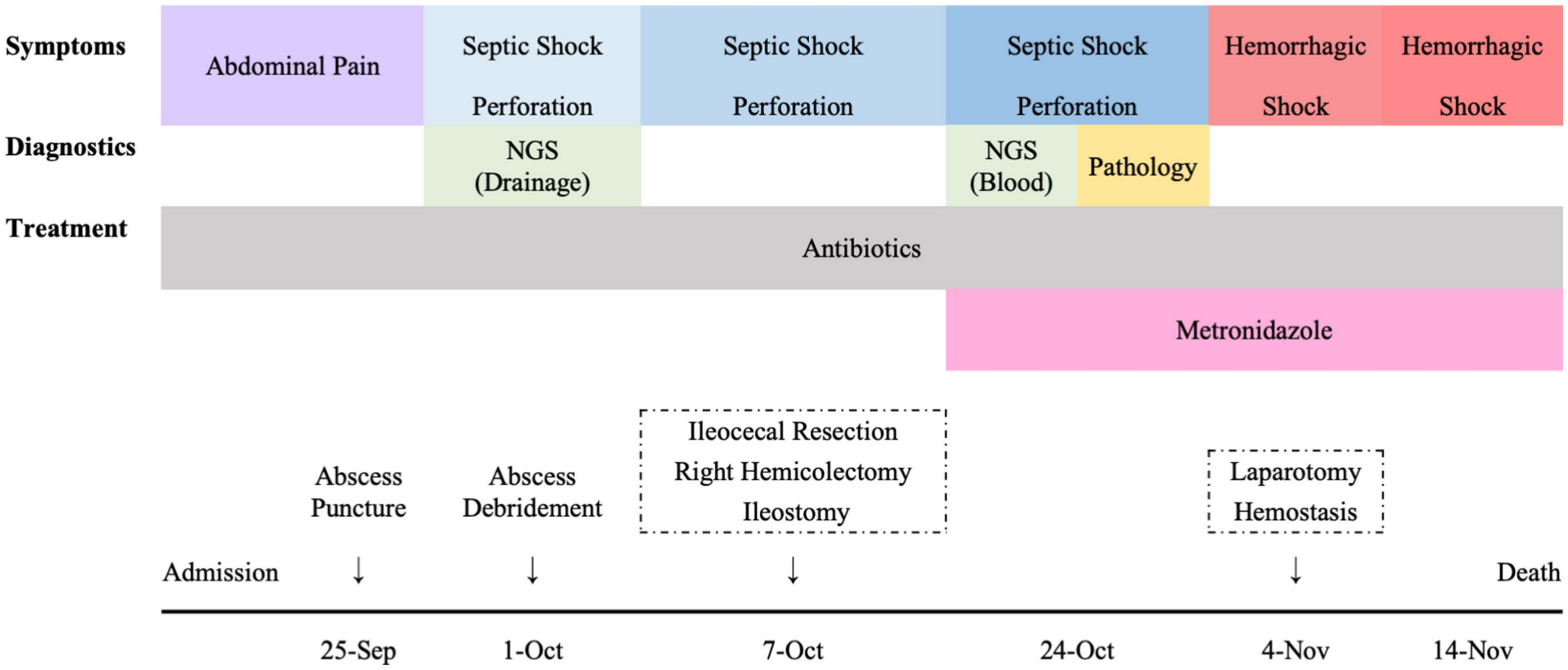
A timeline of the diagnostic and therapeutic course.

A timeline of the diagnostic and therapeutic course is shown in Figure 2.

mNGS was performed by a certified clinical laboratory (Yuguo Biotechnology Co., Ltd., Beijing, China). For each test, 2 mL of ascites fluid or whole blood sample was collected into an EDTA tube and processed within 4 h. Total nucleic acids were extracted using the QIAamp DNA Micro Kit (QIAGEN, Hilden, Germany), and DNA libraries were prepared using Qiagen’s library construction kit (QIAseq Ultralow Input Library Kit). Sequencing was performed on the Illumina550DX platform, generating 150 bp paired-end reads. After removing low-quality reads and sequences aligned to the human reference genome (hg38), the remaining sequences were aligned against a microbial reference database containing bacteria, viruses, fungi, and parasites using BLASTN (v2.10.1+). A pathogen was reported when the read count for a specific species exceeded the laboratory-validated reporting threshold and was consistent with the clinical presentation. The turnaround time from sample receipt to final report was approximately 16 h.

## Discussion

Amebiasis remains a substantial global health burden, with the highest prevalence in low-income tropical and subtropical regions where socioeconomic conditions and sanitation infrastructure are limited (1, 2). In contrast, in high-income countries most cases occur in immigrants, travelers, or other individuals returning from endemic areas (3).

The clinical spectrum of amebiasis is broad, ranging from mild abdominal discomfort and diarrhea to severe complications, such as liver abscess, peritonitis, and septic shock. Some patients are diagnosed only after life-threatening events, such as abscess rupture, fulminant colitis, or hemorrhagic shock (4), and the disease is frequently misdiagnosed as a bacterial abscess or other infectious colitis, delaying targeted therapy. The present case illustrates the complete pathogenic trajectory from subclinical intestinal infection to perforation, septic shock, and ultimately fatal intra-abdominal hemorrhage. It underscores the diagnostic challenges in non-endemic settings and the pivotal role of mNGS when conventional modalities are unavailable or yield inconclusive results.

For patients with a history of exposure, valuable information can be obtained using PCR and serological testing with appropriate samples. However, the application of these methods in clinical practice may be limited, for example, by the need for a certain level of clinical suspicion and the limited availability of testing methods in hospitals in non-endemic areas (5). Histopathology remains the gold standard for diagnosis, but it is invasive and can usually only be performed after surgery or biopsy. In contrast, mNGS can be applied to a variety of clinical samples and does not rely on pre-selected targets, thus offering diagnostic value in complex or atypical cases.

mNGS offers major advancements in the diagnosis of parasitic diseases. Its unbiased, high-sensitivity, and multipathogen detection capabilities enable the identification of *E. histolytica* with atypical presentations, mixed infections, and cases with negative conventional tests (6). mNGS relies on the metagenomic sequencing of all nucleic acids within a sample, followed by bioinformatic alignment to species-specific genomic signatures. The organism’s distinct genome (~20.8 Mb) provides a robust reference for precise identification (7).

Currently, most reports on the use of mNGS for *E. histolytica* diagnosis focus on extraintestinal infections, such as hepatic abscesses, whereas its clinical application in intestinal amebiasis remains at an early stage. Multiple studies have used 18S rRNA sequencing or shotgun metagenomic sequencing to detect intestinal protozoa in stool samples. However, to our knowledge, there are currently no case reports demonstrating the use of fecal mNGS as a primary clinical diagnostic tool for amoebic colitis. In current clinical practice, fecal antigen testing and species-specific PCR remain the first-line diagnostic methods for suspected intestinal amoebiasis. Due to the large amount of background bacteria and host nucleic acids in stool, the incremental diagnostic value of non-targeted NGS compared to multiplex stool PCR is uncertain. In contrast, in our case, mNGS testing of ascites fluid (contaminated with intestinal contents during intestinal perforation) yielded a highly reliable signal for *E. histolytica*, and this result was obtained before histopathological confirmation. In patients with intestinal amebiasis, trophozoites can be detected within the intestinal lumen or wall, and in cases of bowel perforation, amebic DNA may spill into the peritoneal cavity along with intestinal contents. More importantly, trophozoites can disseminate hematogenously to other tissues or organs, leading to more extensive disease. Furthermore, the trophozoite stage lacks a cell wall and is enclosed only by a cell membrane; thus, its nucleic acids can be readily released and efficiently captured by mNGS workflows. These features provide both the rationale and technical feasibility for applying mNGS in the diagnosis of intestinal amebiasis.

In the diagnosis of amoebiasis, the interpretation of mNGS reads is particularly important. These reads represent short DNA or RNA fragments generated from nucleic acids in the sample. Published cases (Table 1) have shown significant differences in read counts across different sample types: cerebrospinal fluid samples may yield tens of thousands of reads, while serum samples typically yield only tens to hundreds of reads. Therefore, the number of reads is not linearly correlated with the pathogen load. The read counts must be interpreted in conjunction with the sample type, pre-test probability, and clinical severity. Amebic trophozoites degrade rapidly in blood; therefore, even negligible numbers of high-quality reads in high-risk patients may indicate true invasive infection (8). Similarly, the peritoneal fluid obtained after intestinal perforation may contain organismal DNA directly linked to the infectious focus (9). In our patient, the first episode of bowel perforation (October 1, 2025) yielded a peritoneal fluid mNGS positive for *E. histolytica* (19 reads, 80.12% abundance); however, this finding did not initially trigger anti-amebic therapy. After the third perforation, when blood mNGS detected *E. histolytica* (12 reads, 73.71% abundance), metronidazole administration was initiated. Whether earlier treatment based solely on initial mNGS results may have altered the disease trajectory deserves careful reflection.

**Table 1:** Reported studies using mNGS for the diagnosis of amebic infection and comparison of read counts across different sample types.

Year	Country	Diagnosis	Sample	Reads	Comparator
Feng et al. (21) (2025)	China	BAE	CSF	495,127	CT, MRI, Biopsy
			Serum	4	
Lin et al. (22) (2024)	China	PAM	CSF	10,314	CT
			Serum	2,503	
Li et al. (23) (2024)	China	BAE	CSF	724	MRI
Qin et al. (24) (2024)	China	BAE	CSF	2,720	CT, MRI
Li et al. (25) (2023)	China	Liver abscess	Drainage	Negative	CT, MRI, PET-CT, Biopsy
			Abscess fluid	81	
Che et al. (26) (2023)	China	PAM	CSF	23,834	CT
Liu et al. (27) (2023)	China	BAE	CSF	25,386	CT, MRI, Biopsy
Wang et al. (9) (2022)	China	Liver abscess	Drainage	Negative	Contrast-enhanced CT
			Exudate (center/ near wall)	5/306	
Xu et al. (28) (2022)	China	BAE	CSF	399	CT, MRI, PET-CT
Zhou et al. (29) (2022)	China	PAM	CSF	42,899	CT, MRI, PCR, Microscopy
			Serum	3,025	
Hu et al. (30) (2022)	China	BAE	CSF	209	MRI
Peng et al. (31) (2022)	China	BAE	CSF	539	MRI
			Serum	112	
			Tissue	3,723	
Ai et al. (32) (2021)	China	BAE	CSF	150	MRI, PCR, Microscopy
Huang et al. (33) (2021)	China	PAM	CSF	326	MRI
			Serum	64	
Hirakata et al. (34) (2021)	Japan	BAE	Tissue	129	MRI, PCR, Microscopy
Yang et al. (35) (2020)	China	BAE	CSF	1,640	CT, MRI

CSF, cerebrospinal fluid; BAE, Balamuthia amoebic encephalitis; PAM, primary amoebic meningoencephalitis.

The patient initially presented with an appendiceal abscess, which rapidly progressed to bowel perforation, diffuse peritonitis, and septic shock. Management centered on pathogen-directed therapy, surgical intervention, and organ support.

Metronidazole (or other nitroimidazoles) is the first-line therapy for invasive amebiasis that targets tissue-dwelling trophozoites. Given the impaired gastrointestinal function and septic physiology, IV administration is appropriate. Since metronidazole does not eradicate intraluminal cysts, a luminal agent such as paromomycin is recommended after clinical stabilization to prevent relapse and transmission (10). In this case, the patient’s condition deteriorated too rapidly to complete the second stage.

Fulminant amebic colitis is characterized by friable intestinal tissue and extensive vascular destruction, making primary anastomosis hazardous owing to the risks of anastomotic failure and postoperative hemorrhage (11). Based on imaging and operative findings, resection of the necrotic bowel, creation of a stoma, and extensive peritoneal lavage and drainage were performed (12). Despite aggressive surgical and antimicrobial management, recurrent intra-abdominal bleeding ultimately results in fatal hemorrhagic shock, reflecting progressive amebic vascular injury.

*E. histolytica* has a biphasic life cycle comprising of cysts and invasive trophozoites, and the transition between these stages is central to pathogenicity (13). Trophozoites adhere to the intestinal epithelium via the Gal/GalNAc lectin and secrete cytotoxic effectors, including cysteine proteases and amebapores, which disrupt epithelial integrity and create superficial erosion (14). These virulence factors also degrade the mucosal layer, exposing the lamina propria and enabling deeper invasion. Once the mucosal barrier is breached, trophozoites penetrate the submucosa and produce the characteristic “flask-shaped ulcers,” which contain necrotic debris, trophozoites, and inflammatory cells (15). Such ulcers often abut or expose substantial submucosal vessels whose walls are markedly weakened by inflammatory injury (16). Continued invasion of the muscularis and serosa leads to transmural necrosis and perforation, as was observed intraoperatively in this case (17). At this stage, intramural vessels may be eroded or directly invaded, predisposing patients to catastrophic hemorrhage.

Additionally, amebic infection triggers robust innate and adaptive immune activation (18), with high levels of pro-inflammatory cytokines promoting endothelial dysfunction, coagulation factor consumption, fibrinolytic overactivation, and thrombocytopenia. These coagulation abnormalities further exacerbate the bleeding risk (19, 20). In this patient, markedly elevated IL-1β, IL-6, IL-8, and IL-10 levels and reduced IgG/IgM and complement C3/C4 supported profound immune dysregulation consistent with severe invasive amebiasis.

## Conclusion

This case of *Entamoeba histolytica* colitis complicated by appendiceal perforation, septic shock, and fatal intra-abdominal hemorrhage underscores the diagnostic and therapeutic challenges of invasive amebiasis in a non-endemic setting. This case highlights that in non-endemic regions, mNGS may serve as a sensitive and rapid tool for detecting intestinal amebic infections, facilitating the early initiation of anti-amoebic therapy. Clinicians should remain vigilant of amebiasis-associated intra-abdominal bleeding and consider timely surgical interventions to improve patient outcomes.

## Data Availability

The raw data supporting the conclusions of this article will be made available by the authors, without undue reservation.
